# Moral dilemmas of community pharmacists: a narrative study

**DOI:** 10.1007/s11096-017-0561-0

**Published:** 2017-11-20

**Authors:** Martine Kruijtbosch, Wilma Göttgens-Jansen, Annemieke Floor-Schreudering, Evert van Leeuwen, Marcel L. Bouvy

**Affiliations:** 1 SIR Institute for Pharmacy Practice and Policy, Leiden, The Netherlands; 2 Radboud University Medical Center, Radboud Institute for Health Sciences (RIHS), Scientific Center for Quality of Healthcare (IQ Healthcare), Nijmegen, The Netherlands; 3 Division of Pharmacoepidemiology and Clinical Pharmacology, Department of Pharmaceutical Sciences, Utrecht University, Utrecht, The Netherlands

**Keywords:** Community pharmacists, Moral dilemmas, Netherlands, Pharmacy ethics

## Abstract

*Background* Pharmacists are increasingly involved in patient care. This new role in a complex healthcare system with demanding patients may lead to moral dilemmas. There has been little research into pharmacy ethics, and existing data are limited by their retrospective nature and small sample sizes. A thematic overview of the moral dilemmas experienced by community pharmacists is still missing. *Objective* To make a thematic overview of moral dilemmas experienced in daily pharmacy practice. *Setting* Dutch community pharmacy. *Methods* Dutch community pharmacists wrote a narrative about a moral dilemma they had experienced in clinical practice. The narratives were analysed using qualitative content analysis to identify underlying themes. *Main outcome measure* Themes of moral dilemmas. *Results* Twenty-two themes were identified in 128 narratives. These moral dilemmas arose predominantly during pharmacists’ contact with patients and other health professionals. The relationship between the pharmacist, patient and other health professionals was complicated by other parties, such as legal representatives, health insurance companies, and regulators. *Conclusion* The moral dilemmas experienced by community pharmacists are more diverse than previously reported. The main dilemmas arose in their professional contacts, frequently when their professional autonomy was challenged by the behaviour of patients and other health professionals.

## Impact on practice

- The moral dilemmas for pharmacists are diverse, but underlying is often a troubled relationship with physicians and/or patients that hampers the pharmacist to deliver appropriate pharmaceutical care.
- Reflection on moral dilemmas may help community pharmacists to strengthen their professional autonomy.
- Investing in good professional relationships with other health professionals and in a therapeutic relationship with patients may benefit recognition of pharmacists’ expertise.

## Introduction

 Worldwide, the primary focus of pharmacists is shifting from products to patients [1–7]. This patient-centred approach means that pharmacists have to identify patients’ concerns and needs, and collaborate closely with other health professionals in order to ensure effective and safe use of medicines [8]. Lastly, pharmacists are responsible for helping patients to achieve definite health outcomes.

Nowadays, health professionals such as pharmacists are faced with ever more regulations, financial pressure, and increased competition. At the same time, the demand for health services is growing as a result of population ageing, more chronic illnesses, and increased healthcare consumerism [9, 10]. Economic and legal constraints and demanding patients challenge health professionals’ autonomy to act in the best interests of society and the individual patient [9, 11–19]. In this complex setting, pharmacists are frequently confronted with *moral* dilemmas [20–30] arising from conflicting personal, professional, institutional or societal values of the different parties involved [28, 31–33].

These moral dilemmas need to be studied in order to address the challenges pharmacists face in their professional role [24, 29, 30, 34–39].

There have been few international studies of the moral dilemmas experienced by community pharmacists, and existing studies vary widely in aim, method and presentation of results [24–27, 29, 30, 40]. In most existing studies, pharmacists were presented with scenarios of moral dilemmas and their moral reasoning was assessed. Pharmacists found it difficult to recall moral dilemmas and most studies interviewed a limited number of pharmacists [20–23, 30, 41]. Hence the themes of moral dilemmas experienced in clinical practice may still be incomplete.

## Aim of the study

We aimed to make a thematic overview of moral dilemmas community pharmacists actually experienced in clinical practice.

### Ethics approval

The Medical Ethics Review Committee of the University Medical Centre Leiden concluded that the Dutch Medical Research Involving Human Subjects Act (WMO) was not applicable. All participants consented that their narratives could be used for the purpose of the study. Data that could give clues about the origin of dilemmas (e.g. names of patients, cities, pharmacies, pharmacists or physicians) were removed.

## Methods

### Study design and setting

Pharmacists wrote a narrative of a moral dilemma they had experienced in clinical practice, as an assignment during either pre- or postgraduate training. The pharmacists were asked to write this narrative immediately after they had experienced the dilemma. A stratified random sample of these narratives was taken. All pharmacists had been taught how to recognise moral dilemmas. This study followed scientific standards for reporting qualitative research (SRQR; see “Appendix”) [42, 43].

### Definition of moral dilemmas

On the basis of the various definitions in the literature [25, 29, 35, 40, 44, 45], a ‘moral dilemma’ was defined as: *a situation in which there is a choice between at least two courses of action, neither of which is obviously morally preferable*. Narratives were checked against this working definition by both the first author (MK) and a member of an expert panel consisting of eleven senior pharmacists active in the special interest group on pharmacy ethics of the Royal Dutch Pharmacists Association. All panellists had been trained in a half-day ethics course to identify moral dilemmas. If consensus was not reached, a third pharmacist from the research group (WG, MB or AF) was consulted. Narratives that did not comply with our working definition were excluded.

### Data analysis

Inductive content analysis of narratives to identify themes of moral dilemmas was facilitated with ATLAS.ti (version 7.5.17) [46]. Consensus on final themes and main categories was reached in two rounds, during independent validation by the research group and during a consensus meeting with both the expert panel and research group.

## Results

**Fig. 1:**
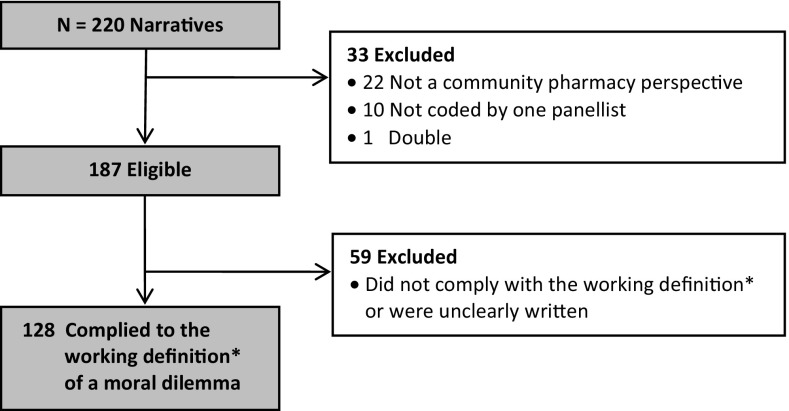
Inclusion of narratives. *A situation in which there is a choice between at least two courses of actions, neither of which is obviously morally preferable

Of the 220 narratives, 92 were excluded (Fig. 1). The included 128 narratives were written by pregraduates (49%: 51% male, 49% female) and postgraduates (51%: 39% male, 61% female). Twenty-two themes were identified, divided into three main categories (see Table 1). Below, we illustrate the themes with a brief summary of a dilemma and quotes from pharmacists that reflect the essence of the theme.

**Table 1:** Themes of moral dilemmas experienced by community pharmacists in clinical practice (N = 128)

***Category 1: The pharmacist–patient relationship***	*n* *=* *59*
• Drug abuse or addiction	10
• Drug misuse	6
• Deviating treatment preference	10
• Claiming and/or aggressive behaviour	7
• Medication understanding	6
• Patient’s privacy	6
• Sharing relevant patient data with health professionals	5
• Public health policy and third-party payer regulations	9
***Category 2: The pharmacist-colleague relationship***	*n* *=* *35*
• Disruptive behaviour of a physician^a^	9
• Disruptive behaviour of a colleague	5
• Pharmacist and physician have a different opinion about appropriate pharmacotherapy	7
• A troubled relationship with the physician	4
• Deviating from a prescription with the physician^a^ absent	3
• Missing relevant patient data with the physician^a^ absent	3
• Loyalty conflicts	3
• Physician’s^a^ self-prescribing	1
***Category 3: Various relationships or involved parties***	*n* *=* *34*
• Reimbursement for a pharmaceutical product or pharmaceutical care	9
• Risk of harm to children	7
• Risk to the unborn child	5
• End-of-life pharmaceutical care	6
• Dispensing without a prescription	5
• Quality defects	2

^a^In this study dentists were also grouped as physicians

### Category 1: The pharmacist–patient relationship

#### Drug abuse or addiction

Patients who had (or were suspected to have) drug abuse or addiction problems requested for refills too soon. The drugs involved were mainly controlled drugs such as opioids or benzodiazepines. Pharmacists grappled with the problem of possibly sustaining addictive behaviour on the one hand and the need to retain patients’ trust and to relieve pain or anxiety on the other.

PHARM-276: This patient is heavily addicted to an opiate and regularly asks for, and gets, a refill too early. “He always has excuses like ‘I carried a heavy load yesterday’ or ‘I lost my medication during holidays’. However, he does experience pain and needs an analgesic. If he needs the opiate because he has really lost it, he should get it. But how long should I contribute to his opiate addiction.”

#### Drug misuse

Drug misuse can lead to side effects or affect the effectiveness of the prescribed medicine. Pharmacists expressed concern about confronting patients without losing their trust. Dispensing *without* addressing the issue also had drawbacks.

PHARM-1082: The pharmacist is aware that a student has been collecting methylphenidate only twice a year (coinciding with exams in January and June). The prescriber is the patient’s father. “Should I cooperate, give priority to the patient’s autonomy and dispense this medicine when I doubt whether the drug is actually indicated for a ‘chronic’ illness? Or do I need to address this presumed off-label use?”

#### Deviating treatment preference

Patient treatment preferences might not be supported by evidence-based medicine or professional guidelines. In these situations, the pharmacists considered patients’ preferences potentially ineffective or harmful.

PHARM-1235: A mother requested paracetamol for her 1-year-old baby who had a high fever for a week. “I tried to convince her to consult her GP as her baby might have a dangerous infection. She didn’t agree. Her attitude frustrated me. I want to do what is best for the baby, but at the same time have to respect the mother’s decision.”

#### Claiming and/or aggressive behaviour

Claiming or aggressive behaviour of patients undermines the trust-based relationship pharmacists have with patients and frustrates pharmacists because it might prevent them from providing adequate care. This is a complex situation, especially if there are other patients waiting in the pharmacy.

PHARM-1062: A patient asked the pharmacist for a prescription of oxazepam that had been faxed 2 months ago. The electronic patient record, however, suggested that the prescription had already been dispensed. The patient became furious and insisted that he had never received the drug and needed it urgently. “I felt attacked, but also had my doubts because of his convincing manner of speaking. Did we make a mistake? Should I dispense once again without a prescription?”

#### Medication understanding

Patients (or their carers) who had difficulties understanding drug information because of language deficiency or limited health literacy, posed a particular problem. Pharmacists doubted whether these patients would use the drug safely, but *not* dispensing was not an option if the patient clearly needed treatment.

PHARM-314: An elderly Spanish speaking patient did not understand the pharmacist who explained the need for gastroprotection during NSAID use. She kept repeating that she was in pain and only needed the NSAID. This situation did not change even with a translator. “I wanted to assist and advise her correctly but poor communication made that impossible. I had my doubts about dispensing the NSAID without gastroprotection because of the possible health risks.”

#### Patient’s privacy/sharing relevant patient data

Sharing patient data with either health professionals or informal carers may be necessary from a clinical perspective. Dilemmas occurred when the pharmacist felt a need to share data, but patient’s consent to share data was absent or patients even requested not to share these data.

PHARM-252: A woman treated for a bipolar disorder told her pharmacist that she wanted to discontinue mirtazapine. She explicitly asked the pharmacist not to notify her psychiatrist. “I explained to her that I couldn’t provide proper pharmaceutical counselling because I didn’t have relevant background information.” Although the pharmacist wanted to respect her autonomy, he also felt he should notify the psychiatrist.

#### Public health policy and third-party payer regulations

In general, current health policy is directed at curbing increasing health expenditure. Dutch health insurance companies reimburse only generic products unless the physician has medical reasons for prescribing a branded drug. While pharmacists recognised their responsibility to reduce health expenditure, this also disturbed their relationship with patients who strongly objected to generics.

PHARM-84: A patient, objected strongly to generic salbutamol. “After persistently trying to explain the drug reimbursement policy to him, I convinced him to try the generic for at least 14 days. A few hours later, he reported numerous complaints. Later the patient came with a prescription for the branded aerosol and a statement from his physician declaring the necessity of him having the original drug. Somehow, I felt the patient had never really tried the generic.” The pharmacist had doubts about whether he should start the conversation with the patient again or fill the prescription.

### Category 2: The pharmacist–colleague relationship

#### Disruptive behaviour of a physician/a troubled relationship with the physician

Pharmacists described situations in which the relationship with physicians was troubled. Sometimes physicians even behaved disruptively e.g. by not listening to the pharmacists’ pharmacotherapy suggestions. This deprived the pharmacists of relevant information and caused frustration because their expertise was not appreciated. Pharmacists had reservations about the safety or effectiveness of prescribed treatment. Not dispensing, however, was equally problematic because reasons for the chosen treatment might have been valid. Moreover, pharmacists were anxious to further disrupt their professional relationship with the physician.

PHARM-54: A cardiologist deviated from the guideline for combining antiplatelet drugs. When the pharmacist requested clarification, the cardiologist’s replied: “Do you mind if I continue with my patients now?” The pharmacist could not properly inform the patient about his doubts about the therapy. “I had strong doubts about the safety of this combination. Informing the patient about the risks, however, might worry the patient and undermine his confidence in the cardiologist.”

#### Disruptive behaviour of a colleague

Pharmacists reported disruptive behaviour of colleagues, such as gossip, lying or suspected fraud. Both neglecting and addressing such behaviour could influence the work climate in the pharmacy.

PHARM-1084: A pharmacy is reimbursed for every patient who receives instructions about a new inhaler. The senior pharmacist asked a junior pharmacist to send a list of all patients who had received a new inhaler with instructions to the insurance company for reimbursement. The junior pharmacist was reluctant to do this, struggling with going against his senior colleague’s request as well as his responsibility to society. “I was uncertain whether the instructions had always been given. Technicians didn’t always document this and patients sometimes refused the instructions or had already received them elsewhere.”

#### Pharmacist and physician have a different opinion about appropriate pharmacotherapy

In these dilemmas physicians ‘overruled’ pharmacists’ proposals, although not necessarily in a brusque manner. Pharmacists had the idea that the physician did not really consider their suggestions and doubted the suboptimal or unsafe pharmacotherapy. Pharmacists felt at a disadvantage because they lacked sufficient knowledge about the patient’s condition. Moreover pharmacists did not want to further disrupt their professional relationship with the physician.

PHARM-22: The pharmacist had suggestions about alternative therapy options for a patient with serious pain complaints. However, the physician said that he had tried everything and that nothing more could be done and did not want to change the medication. “In the end, it is the physician who prescribes. I wanted to help the patient but suggesting these options directly to the patient also did not feel appropriate.”

#### Deviating from a prescription or missing relevant data with the physician absent

Pharmacists had a moral dilemma when they wanted to deviate from a prescription because of potential drug related problems such as interactions or allergy warnings, or to discuss the treatment because lack of relevant clinical data, but could not contact prescribers. Both situations impeded their judgement on the appropriateness of pharmacotherapy.

PHARM-350: A dentist prescribed amoxicillin. The pharmacist knew that the patient had previously had an allergic reaction on amoxicillin. The dentist could not be reached, but the patient urgently needed medication. “What if the dentist does not agree with the alternative antibiotic clindamycin?

#### Loyalty conflicts

Pharmacists had a conflict of loyalty when their decisions would either affect their professional relationship with colleagues or result in suboptimal patient care.

PHARM-115: A physician asked the pharmacist to urgently prepare a midazolam infusion to start palliative sedation for a patient registered at a neighbouring pharmacy. The physician explained that the pharmacist of that pharmacy was not able to prepare the infusion that day. “In my opinion not dispensing wasn’t an option because of the condition of the patient. On the other hand, I didn’t want to overrule the decision of my colleague-pharmacist who is the responsible professional for this patient.”

#### Physician’s self-prescribing

Although this is a well-known issue [47], only one case of physician self-prescribing was reported:

PHARM-1176: A physician prescribed midazolam for himself. “Dispensing felt problematic because sleep medication might have negative effects on the physician’s daily functioning. Moreover, the pharmacist did not want to become the accomplice of an addicted physician. However, not dispensing could damage the professional relationship and future collaboration.

### Category 3: Various relationships or involved parties

The previous categories of dilemmas involved patients or health professionals. In the following themes other ‘stakeholders’ were involved, such as health insurance companies and manufacturers. We also included dilemmas with (unborn) children and adolescents in this category, as pharmacists in these situations have a complex responsibility towards these unborn children, minors and their parent(s) or legal representatives.

#### Reimbursement for a pharmaceutical product/care activity or additional service

Pharmacists experienced dilemmas when patients were not insured and not able to pay their medication out of pocket, because these patients needed their medicines. Also, concerns about pharmacy workload sometimes conflicted with pharmacists’ wish to deliver optimal but time-consuming patient care. Providing additional services for some patients would mean compromising on other services.

PHARM-278: A nursing home requested multidose drug dispensing systems for every patient. This would include anticoagulant medication, the dosing of which often has to be adjusted. The pharmacy did not have the capacity to change the multidose drug dispensing systems manually each week. “I realise the importance of this request, but it would almost take an extra technician without getting any reimbursement.”

#### Risk of harm to (unborn) children

Pharmacists confronted with off-label prescriptions for children and adolescents felt they could not appropriately assess the risk–benefit ratio or the correct dosing of drugs. Another dilemma was when children collected medication. Pharmacists worried about the possibility of the child misusing the medication, but also did not want the patient to be left without medication. Even more complicated moral dilemmas arose when medication was prescribed to pregnant women. In these cases, pharmacists had to weigh the benefits for the mother against the potential risks for the unborn child.

PHARM-1202: “A psychiatrist told me he did not want to tell a pregnant woman with a major depressive disorder about the teratogenic risks of paroxetine because he was afraid that she would not take the drug. The psychiatrist considered that the mother not taking paroxetine would potentially be riskier for the unborn child than the small teratogenic risk. I struggled with appropriate counselling.”

#### End-of-life pharmaceutical care

These dilemmas concern euthanasia or palliative sedation. Dutch Pharmacists’ and Physicians’ Associations have a joint guideline on providing euthanasia [48]. Sometimes physicians did not adhere to the guideline recommendations; e.g. a physician requested euthanasia drugs without timely communication with the pharmacist. Pharmacists were then reluctant to cooperate. However *not* dispensing felt wrong because the patient was suffering.

The dilemmas that dealt with palliative sedation concerned both disagreement about the dose of palliative sedation and the expectations of physicians that pharmacists would have the necessary drugs readily available.

PHARM-57: This pharmacist did not dispense drugs for euthanasia because of religious objections. Surrounding physicians knew about this. A physician from another area, unaware of the objections, requested these drugs too late in the day to find another pharmacist. “Should I remain faithful to my personal values but then trouble both the patient and physician, or should I dispense the drugs this one time?”

#### Dispensing without a prescription

Patients regularly requested (restricted) medicines without a (valid) prescription. In these situations, pharmacists had to balance the necessity and risks of dispensing. Pharmacists felt it hard to make this balance because they had insufficient clinical information and were reluctant to deviate from laws and regulations.

PHARM-71: The middle-aged son of an elderly patient visited the pharmacy just before closing time. He showed a picture on his mobile phone of an oxycodone prescription for his father who had just been discharged from hospital. He said his father suffered from severe pain and he could not get the real prescription in time before the pharmacy closed. “The prescription does not comply with the law but this patient could suffer unnecessarily if I don’t dispense.”

#### Quality defects

These moral dilemmas were related to uncertainty about the quality of a pharmaceutical product and the risks of dispensing a product that might be ineffective or harm patients.

PHARM-309: A patient visited the pharmacy with three golimumab injections worth €3500 which had been outside the refrigerator for about 1 day. “The manufacturer told me they expected no quality issues but could not give any guarantee.” The pharmacist doubted whether the patient would be harmed by using the injections, and felt that, given their cost, discarding the injections was not socially responsible.

## Discussion

This study presents moral dilemmas experienced by community pharmacists in clinical practice. The underlying themes address the challenges pharmacists face while providing care in a complex setting with economic and legal constraints, demanding patients and limited professional autonomy. Analysis showed that most moral dilemmas concerned the relationship between pharmacists, patients and physicians. This is not surprising considering that pharmacists are responsible for helping patients achieve positive health outcomes, and this responsibility requires that they work with patients and other health professionals [49].

As far as we know, no previous study used narratives to understand the moral dilemmas that pharmacists experience in clinical practice [26, 29, 40]. Writing a narrative shortly after a dilemma occurred avoids recall problems and enables pharmacists to reflect directly on their feelings. Previous studies generally interviewed pharmacists and asked them to recall dilemmas that occurred in the past [29, 40]. This may be the reason why, in those studies, pharmacists mainly recalled dilemmas with a high legal impact. For example, pharmacists expressed fear of breaking the law when a patient asked for a controlled drug without a (valid) prescription [26, 29, 40]. When legal issues occurred in this study, pharmacists were more concerned about the patient’s well-being and the mutual trust in the treatment relationship than about breaking the law.

Pharmacists experienced dilemmas during their professional contacts because the behaviour of patients and physicians made it difficult for them to act autonomously, according to their professional core values [50]. Since the days of Hippocrates, health professionals’ core value is not to harm patients and to act in their best interest. However, conflicts may arise when more than one health professional aims to act according to that value. The degree of professional autonomy of an individual health professional depends on the extent to which other health professionals grant that autonomy [51]. Regular collaboration between pharmacists and other health professionals may promote mutual trust and respect for each other’s knowledge and expertise [52–54].

Pharmacists’ autonomy may also be challenged because pharmacists are often the last link in a multidisciplinary care chain, e.g. in *end*-*of*-*life pharmaceutical care* issues. In that position pharmacists’ expertise comes into play too late or is not recognised [55, 56]. Pharmacists in these situations described that their expertise was disregarded and that they were expected to dispense only. These moral dilemmas demonstrate that pharmacists need more training to convince physicians of their expertise.

The professional autonomy of pharmacists may also be restricted by patients or parties such as insurance companies or the health inspectorate. Patients may also consider physicians to have more authority than pharmacists. This can sometimes lead to *claiming and/or aggressive behaviour* of patients. This behaviour undermines the trust relationship between the pharmacist and the patient. This resembles healthcare consumerism, which is reported to challenge the ability of health professionals to optimally fulfil patients’ and societal needs [9–14]. Dilemmas under the theme *public health policy and third*-*party payer regulations* showed that health insurance companies can also undermine pharmacists’ autonomous professional decision-making and actions. Insurance companies oblige pharmacists to replace expensive branded drugs with cheaper generics. Although pharmacists do not object to dispensing cheaper medicines whenever possible, this responsibility also disturbed their relationship with patients who strongly objected to generics. This finding confirms a worldwide trend that economically motivated health policies challenge the professional autonomy of all health professionals [12–14, 17, 18]. Health policy makers should realise that weakening health professionals’ autonomy, for example due to reimbursement policies, may negatively affect patients’ trust in health care [19].

### Limitations

This study has some limitations. Firstly, the moral dilemmas were reported by ‘early career’ pharmacists. These pharmacists may be more committed to patients’ well-being, because of more advanced training on the patient perspective than earlier generations of pharmacists. Moreover, the training provided might have influenced their sensitivity for moral dilemmas [57]. The themes underlying the moral dilemmas were not less numerous than those in previous studies involving more experienced pharmacists. Exceptions to this are primary business dilemmas. The underreporting of these types of dilemmas might be explained by the fact that the early career pharmacists in our study generally do not own a pharmacy. A second limitation is that saturation of themes was not formally assessed. We did, however, have no clues on additional themes from the excluded narratives. Moreover, screening of an additional stratified random sample of 50 narratives by two authors (MK and MB) neither gave clues on missed themes. Therefore, we are of the opinion that the most important themes for the Dutch context are identified. This does, however, not imply that every individual pharmacist will identify these dilemmas. Furthermore, our results are not completely generalizable to countries with different health systems and a different position of the community pharmacist in health care. Lastly, the written narratives contained much richer information than reported in our brief summaries; some narratives were excluded because they were unclear. As we were primarily interested in the themes, we did not analyse the feelings of pharmacists in depth.

### Implications for practice

This study suggests that a short training enables pharmacists to write narratives on moral dilemmas they experience in clinical practice. Reflecting on these dilemmas may help pharmacists to increase their professionalism. Hence, we suggest to integrate such ethical training in experiential learning within both pre- and postgraduate education. This will raise pharmacists’ awareness on moral conflicts and will support the profession’s transition to delivering pharmaceutical care.

## Conclusion

Pharmacists experience a number of moral dilemmas in clinical practice. The narrative method enables pharmacists to reflect directly on their feelings at the time these dilemmas occur. Most dilemmas involve the pharmacists’ professional relationships and often arise when the professional autonomy of pharmacists is challenged by patients’ and other health professionals’ behaviour.

## Appendix: Standards for reporting qualitative research [42, 43]

**Table:**

Topic	Check	Remarks
Title	X	
Abstract	X	
*Introduction*		
Problem formulation	X	Included in ‘Introduction’
Purpose or research question	X	Included in ‘Introduction’
*Methods*		
Qualitative approach and research paradigm	X	Narrative research. Inductive formulation of themes. The research paradigm was interpretivist/constructivist [43] as predominantly qualitative techniques were used to formulate themes (see ‘Definition of moral dilemmas’ and ‘Data analysis’). Rationale for the qualitative approach was the aim to find themes of moral dilemmas within written narratives
Researcher characteristics and reflexivity	X	Panellists and the research team were all experienced pharmacists and trained in a half-day ethics course to identify moral dilemmas. The first researcher is a sociologist with a pre-master in applied ethics and 12 years work experience in pharmacy practice research
Context	X	The participating pharmacists wrote their narratives as an assignment during either pre- or postgraduate training. They consented to the use of their narratives for research purposes. Only these written narratives were selected and analysed. See ‘Study design and setting’
Sampling strategy	X	Included in ‘Study design and setting’
Ethical issues pertaining to human subjects	X	Included in ‘Ethics approval’
Data collection methods	X	The narratives were written by the participating pharmacists as an assignment during either pre- or postgraduate training
Data collection instruments and technologies		Not relevant as the participating pharmacists wrote the narratives used
Units of study	X	Early career pharmacists
Data processing	X	Included in ‘Ethics approval’ and ‘Data analysis’
Data analysis	X	Qualitative content analysis was used to identify themes (see ‘Data analysis’)
Techniques to enhance trustworthiness	X	Included in ‘Definition of moral dilemmas’, ‘Data analysis’ and ‘Limitations’
*Results/findings*		
Synthesis and interpretation	X	Included in ‘Results’ and ‘Discussion’
Links to empirical data	X	Included in ‘Results’ and ‘Discussion’
*Discussion*		
Integration with prior work, implications, transferability, and contribution(s) to the field	X	Included in ‘Discussion’ and ‘Implications for practice’
Limitations	X	Included in ‘Discussion’
*Other*		
Conflicts of interest	X	Not applicable
Funding	X	Unconditional research grant from the Royal Dutch Pharmacists Association and from the foundation ‘Stichting Management voor Apothekers en voor de Gezondheidszorg’ (MAG)

## References

[1] Gilbert L (1998). Pharmacy’s attempts to extend its roles: a case study in South Africa. Soc Sci Med.

[2] Edmunds J, Calnan MW (2001). The reprofessionalisation of community pharmacy? An exploration of attitudes to extended roles for community pharmacists amongst pharmacists and General Practitioners in the United Kingdom. Soc Sci Med.

[3] van Mil JW, Schulz M, Tromp TF (2004). Pharmaceutical care, European developments in concepts, implementation, teaching, and research: a review. Pharm World Sci.

[4] Wiedenmayer K, Summers RS, Mackie CA, Gous AGS, Everard M, Tromp D. Developing pharmacy practice: a focus on patient care Handbook-2006 edition. World Health Organization (WHO), International Pharmaceutical Federation (FIP). http://www.fip.org/files/fip/publications/DevelopingPharmacyPractice/DevelopingPharmacyPracticeEN.pdf. Accessed 8 Jan 2016.

[5] Hutchings HA, Rapport FL, Wright S, Doel ME, Wainwright P (2010). Obtaining consensus regarding patient-centred professionalism in community pharmacy: nominal group work activity with professionals, stakeholders and members of the public. Int J Pharm Pract.

[6] McDonald R, Cheraghi-Sohi S, Sanders C, Ashcroft D (2010). Professional status in a changing world: the case of medicines use reviews in English community pharmacy. Soc Sci Med.

[7] Mossialos E, Courtin E, Naci H, Benrimoj S, Bouvy M, Farris K (2015). From, “retailers” to health care providers: transforming the role of community pharmacists in chronic disease management. Health Policy.

[8] Hepler CD (1990). Opportunities and responsibilities in pharmaceutical care. Am J Health Syst Pharm.

[9] Hibbert D (2002). Consumerism and professional work in the community pharmacy. Sociol Health Illn.

[10] Stevenson FA, Leontowitsch M, Duggan C (2008). Over-the-counter medicines: professional expertise and consumer discourses. Soc Health Illn.

[11] Pellegrino ED (1994). Patient and physician autonomy: conflicting rights and obligations in the physician–patient relationship. J Contemp Health Law Policy.

[12] Freidson E (2001). Professionalism: the third logic.

[13] Bush J, Langley CA, Wilson KA (2009). The corporatization of community pharmacy: implications for service provision, the public health function, and pharmacy’s claims to professional status in the United Kingdom. Res Social Adm Pharm.

[14] Rapport F, Doel MA, Hutchings HA, Wright S, Wainwright P, John DN (2010). Eleven themes of patient-centred professionalism in community pharmacy: innovative approaches to consulting. Int J Pharm Pract.

[15] Timmermans S, Oh H (2010). The continued social transformation of the medical profession. J Health Soc Behav.

[16] Emanuel EJ, Pearson SD (2012). Physician autonomy and health care reform. JAMA.

[17] Tonkens E (2013). Pretenders and performers: professional responses to the commodification of health care. Soc Theory Health.

[18] Roche C, Kelliher F (2014). Giving, “best advice”: proposing a framework of community pharmacist professional judgement formation. Pharmacy.

[19] FIP. Reference Document. Pharmacist ethics and professional autonomy: imperatives for keeping pharmacy aligned with the public interest. International Pharmaceutical Federation (FIP), 2014. http://www.fip.org/www/uploads/database_file.php?id=358&table_id=. Accessed 14 Jan 2016.

[20] Lowenthal W (1988). Ethical dilemmas in pharmacy. J Med Ethics.

[21] Hibbert D, Rees JA, Smith I (2000). Ethical awareness of community pharmacists. Int J Pharm Pract.

[22] Vitell SJ (1991). The business ethics of pharmacists: conflicts practices and beliefs. J Bus Ethics.

[23] Latif DA (2001). The relationship between pharmacists’ tenure in community setting and moral reasoning. J Bus Ethics.

[24] Wingfield J, Bissell P, Anderson C (2004). The scope of pharmacy ethics-an evaluation of the international research literature, 1990–2002. Soc Sci Med.

[25] Kalvemark S, Hoglund AT, Hansson MG, Westerholm P, Arnetz B (2004). Living with conflicts–ethical dilemmas and moral distress in the health care system. Soc Sci Med.

[26] Chaar B, Brien J, Krass I (2005). Professional ethics in pharmacy: the Australian experience. Int J Pharm Pract.

[27] Cooper RJ, Bissell P, Wingfield J (2007). A new prescription for empirical ethics research in pharmacy: a critical review of the literature. J Med Ethics.

[28] Cooper RJ, Bissell P, Wingfield J (2008). Ethical decision-making, passivity and pharmacy. J Med Ethics.

[29] Benson A, Cribb A, Barber N (2009). Understanding pharmacists’ values: a qualitative study of ideals and dilemmas in UK pharmacy practice. Soc Sci Med.

[30] Deans Z. Ethics in pharmacy practice. Pharmacy Practice Research Trust, 2010. http://www.pharmacyresearchuk.org/waterway/wp-content/uploads/2012/11/Ethics_in_pharmacy_practice_200910.pdf. Accessed 12 June 2016.

[31] Cooper RJ, Bissell P, Wingfield J (2009). ‘Islands’ and ‘doctor’s tool’: the ethical significance of isolation and subordination in UK community pharmacy. Health.

[32] Altilio JV (2009). The pharmacist’s obligations to patients: dependent or independent of the physician’s obligations?. JLME.

[33] Cipolle RJ, Morley PC, Strand LM (2012). Pharmaceutical care practice: the patient-centered approach to medication management.

[34] Weinstein BD (1997). Teaching pharmacy ethics: the case study approach. J Pharm Teach.

[35] Latif DA (2001). The relationship between ethical reasoning and the perception of difficulty with ethical dilemmas in pharmacy students: implications for teaching professional ethics. J Bus Ethics.

[36] Latif DA. An assessment of the ethical reasoning of United States pharmacy students: a national study. Am J Pharm Educ 2004;68:Article 30.

[37] O’Fallon MJ, Butterfield KD (2005). A review of the empirical ethical decision-making literature: 1996–2003. J Bus Ethics.

[38] Scharr K, Bussières JF, Prot-Labarthe S, Bourdon O (2011). A comparative pilot study of the professional ethical thinking of Quebec pharmacy residents and French pharmacy interns. Int J Clin Pharm.

[39] Schlesselman LS (2014). A cross-sectional study of applied bioethical reasoning in pharmacy students and preceptors. Pharm Pract.

[40] Cooper RJ, Bissell P, Wingfield J (2007). Dilemmas in dispensing, problems in practice? Ethical issues and law in UK community pharmacy. Clin Ethics.

[41] Bolt I, van den Hoven M, Blom L, Bouvy M (2015). To dispense or not to dispense? Ethical case decision-making in pharmacy practice. Int J Clin Pharm.

[42] O’Brien BC, Harris IB, Beckman TJ, Reed DA, Cook DA (2014). Standards for reporting qualitative research: a synthesis of recommendations. Acad Med.

[43] Mackenzie N. Research dilemmas: Paradigms, methods and methodology. Issues In Educational Research. 2006;16. http://www.iier.org.au/iier16/mackenzie.html. Accessed 15 June 2016.

[44] Braunack-Mayer AJ (2001). What makes a problem an ethical problem? An empirical perspective on the nature of ethical problems in general practice. J Med Ethics.

[45] De Veer AJE. Morele dilemma’s in het dagelijks werk van verpleegkundigen en verzorgenden. NIVEL, Utrecht: 2009. http://www.nivel.nl/sites/default/files/bestanden/rapport-morele-dilemmas-venv.pdf. Accessed 13 June 2016.

[46] Mayring P. Qualitative content analysis. Forum Qualitative Sozialforschung/Forum: Qualitative Social Research. 2000; 1(2), Art. 20. http://www.qualitative-research.net/index.php/fqs/article/view/1089. Accessed 13 June 2016.

[47] Moberly T (2014). Physician, don’t heal thyself: the perils of self prescribing. BMJ.

[48] KNMG/KNMP. Richtlijn Uitvoering euthanasie en hulp bij zelfdoding, 2012. https://www-knmp-nl.proxy.library.uu.nl/downloads/richtlijn-uitvoering-euthanasie-en-hulp-bij-zelfdoding.pdf. Accessed 13 June 2016.

[49] Worley MM, Schommer JC, Brown LM, Hadsall RS, Ranelli PL, Stratton TP (2007). Pharmacists’ and patients’ roles in the pharmacist–patient relationship: are pharmacists and patients reading from the same relationship script?. Res Soc Adm Pharm.

[50] Kasher A (2005). Professional ethics and collective professional autonomy: a conceptual analysis. Ethical Perspect.

[51] MacDonald C (2002). Relational professional autonomy. Camb Q Healthc Ethics.

[52] Sanchez LT (2014). Disruptive behaviors among physicians. JAMA.

[53] Doucette WR, Nevins J, McDonough RP (2005). Factors affecting collaborative care between pharmacists and physicians. Res Soc Adm Pharm.

[54] Rathbone AP, Mansoor SM, Krass I, Hamrosi K, Aslani P (2016). Qualitative study to conceptualise a model of interprofessional collaboration between pharmacists and general practitioners to support patients’ adherence to medication. BMJ Open.

[55] Lindblad AK, Kjellgren KI, Ring L, Maroti M, Serup J (2006). The role of dermatologists, nurses and pharmacists in chronic dermatological treatment: patient and provider views and experiences. Acta Derm Venereol.

[56] Van C, Costa D, Abbott P, Mitchell B, Krass I (2012). Community pharmacist attitudes towards collaboration with general practitioners: development and validation of a measure and a model. BMC Health Serv Res.

[57] Park M, Kjervik D, Crandell J, Oermann MH (2012). The relationship of ethics education to moral sensitivity and moral reasoning skills of nursing students. Nurs Ethics.

